# Emergency Department Wait Times for Urgent Evaluation by Race, Ethnicity, and Language: A Single-center Retrospective Study

**DOI:** 10.5811/westjem.43480

**Published:** 2025-09-20

**Authors:** Jossie A. Carreras Tartak, Anne V. Grossestreuer, David Chiu, Bryan Stenson

**Affiliations:** Beth Israel Deaconess Medical Center, Department of Emergency Medicine, Boston, Massachusetts

## Abstract

**Introduction:**

Black and Hispanic patients, and patients with a preferred language other than English experience longer emergency department (ED) wait times and delays in treatment. We aimed to evaluate racial, ethnic, and language-based differences in wait times to see a physician and get a disposition, as well as in the rates of objective vs subjective urgent evaluations.

**Methods:**

This was a retrospective study of all ED visits in our tertiary-care, academic medical center from July 2021–June 2023. Using electronic health record data, we compared time-to-physician, physician-to-decision times, and frequency of triggers (urgent evaluations based on objective criteria) and priority assessments (urgent evaluations that can be based on subjective perception of patient acuity) by race, ethnicity, and preferred language. We used logistic regression, controlling for age, Emergency Severity Index, and sex to compare differences in trigger rates.

**Results:**

We included 93,728 patient encounters in this study. Black patients had a median time-to-physician of 31 minutes compared to 24 minutes for White patients (adjusted median difference (aMD) 3.2, 95% CI 2.4–3.9]) and a median physician-to-decision time of 228 minutes compared to 213 for White patients (aMD 15.0, 95% CI 12.0–17.9). Hispanic patients had a median time to physician of 31 (aMD compared to White patients = 3.4, 95% CI 2.4–3.9) and a median physician-to-decision time of 233 minutes (aMD compared to White patients 21.3, 95% CI 17.5–25.2). Patients with a preferred language other than English had a median time-to-physician of 33 minutes compared to 25 in English-preferring patients (aMD 4.6, 95% CI 3.7–5.6) and a median physician-to-decision time of 234 compared to 214 minutes for English-preferring patients (aMD 17.1, 95% CI 13.6–20.7). Black patients were less likely to have a trigger activated relative to White patients (adjusted odds ratio [aOR] 0.88, 95% CI 0.82–0.95). Black patients (aOR 0.72, 95% CI 0.67–0.77), Hispanic/Latino patients (aOR 0.78, 95% CI 0.71–0.86), and non-English-preferring patients (aOR 0.85, 95%CI 0.78–0.92) were less likely to have a priority assessment called compared to White patients.

**Conclusion:**

Black, Hispanic, and patients who prefer non-English language experience delays in time-to-physician and physician-to-decision time. Black patients are less likely to have triggers activated. Black, Hispanic, and patients who prefer non-English language are less likely to have priority assessments activated compared to White patients. These findings underscore the need to develop additional mechanisms for mitigating biases in the triage process.

## INTRODUCTION

Emergency departments (ED) play a critical role in providing timely medical care for patients in acute situations where delays can lead to worse outcomes. However, evidence shows that disparities exist in how quickly patients from different racial, ethnic, and linguistic backgrounds are evaluated in the ED.^1^–^6^ Longer wait times can lead to patients leaving without being seen,^7^ as well as delays in timely diagnosis and treatment of time-sensitive conditions. Racial disparities in time-to-treatment of acute coronary syndrome,^8^ stroke,^9^ and appendicitis^10^ have been documented in the literature. Furthermore, Black and Hispanic patients are more likely to have longer lengths of stay (LOS) in the ED.^11^–^13^ Studies on LOS of patients with limited English proficiency (LEP) have been mixed, with some sites showing longer LOS for patients with LEP.^14^,^15^

While most EDs in the United States use the Emergency Severity Index (ESI) triage algorithm to signal to physicians the acuity of a patient’s condition, several studies have observed racial, ethnic, and language-based disparities in the assignment of ESI triage scores to ED patients with a given chief complaint,^16^,^17^ as well as higher instances of mistriage for Black patients.^18^ Even in hospitals that do not rely on ESI scores, Black and Hispanic patients with a given chief complaint are more likely to be triaged to lower acuity areas in the ED than their White counterparts.^19^

The purpose of this study was to evaluate racial, ethnic, and language-based differences in wait times for a physician and a disposition, as well as in the rates of objective vs subjective urgent evaluations. In this study, we aimed to quantify the following: 1) delays in time to see physician; 2) differences in physician-to-decision time; and 3) differences in the proportion of urgent evaluations across racial, ethnic, and linguistic groups in our own ED. At the time of this study, we used a proprietary electronic health record (EHR), which did not rely exclusively on ESI scores to mandate rapid evaluations of patients with concerning presentations. Furthermore, we employed a system of overhead calls and pages that alerted medical teams about a patient who needed to be urgently evaluated based on vital signs, symptoms, or nursing concerns. We hypothesized that these factors would mitigate the racial, ethnic, and language-based disparities in wait times and LOS reported at other sites. We also hypothesized that rates of urgent evaluations would be similar across all groups.

## METHODS

### Emergency Department Protocols

Our ED uses an overhead and paging alert system for patients requiring urgent evaluation by the medical team. There are two types of urgent evaluation: 1) triggers and rapid assessments; and 2) priority assessments. Both triggers and rapid assessments require a junior and a senior resident to immediately evaluate the patient in question. The main difference between triggers and rapid assessments (besides the specific criteria for activating each) is that attending physicians are technically not required to respond immediately to the rapid assessments, but in practice nearly all of them do. Furthermore, triggers and rapid assessments (unlike priority assessments) are meant to be disruptive and summon clinicians immediately to a patient’s bedside. Because triggers and rapid assessments are in practice treated equally by the medical teams, they are hereby referred to collectively as “triggers.”

**Population Health Research Capsule**

What do we already know about this issue?

*Prior studies have shown that Black, Hispanic, and limited English proficiency (LEP) patients experience longer wait times in the ED*.

What was the research question?

*Do Black, Hispanic, and LEP patients experience longer wait times and lower rates of urgent evaluation?*

What was the major finding of the study?

*Black (aOR 0.72, 95% CI 0.67–0.77), Hispanic (aOR 0.78, 95% CI 0.71–0.86), and LEP patients (aOR 0.85, 95% CI 0.78–0.92) were less likely to get an priority assessment and more likely to wait longer*.

How does this improve population health?

*Our results could lead to interventions like increased outpatient access to care and interpreter availability*.

**Figure 1:**
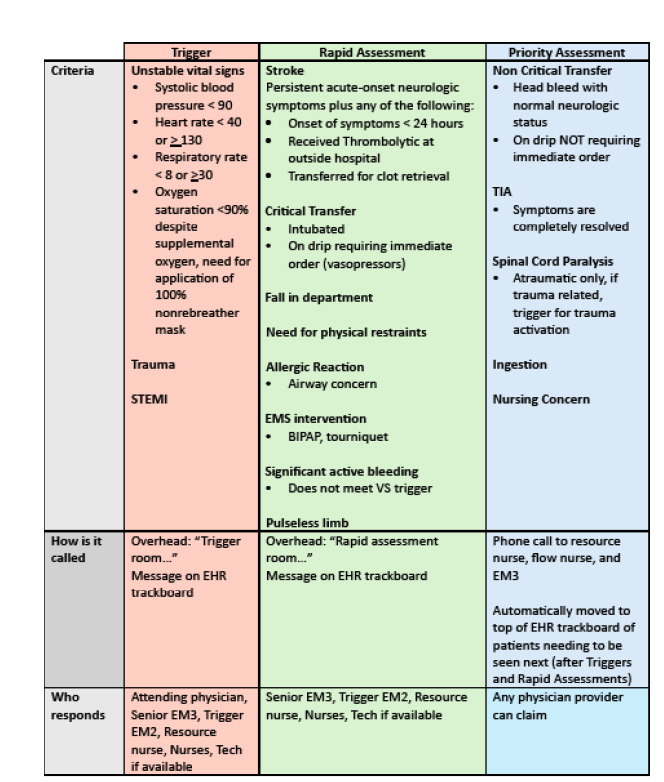
Criteria to activate “trigger,” rapid assessment, and priority assessment in emergency department patients. *BIPAP*, bilevel positive airway pressure; *EHR*, electronic health record; *EMS*, emergency medical services; PGY, postgraduate year; *TIA*, transient ischemic attack; *VS*, vital signs.

When a trigger is announced overhead and via the paging system, a team comprised of a junior and senior resident, and an attending physician must immediately evaluate the patient in question. Triggers can be activated by anyone on the medical team whenever a patient meets certain objective criteria, such as abnormal vital signs, if they are intubated or on positive pressure ventilation, or if they have an immediately life- or limb-threatening chief complaint such as a ST-elevation myocardial infarction, trauma, stroke symptoms, allergic reaction, or pulseless extremity. Alternatively, priority assessments are used to identify patients who should be seen next by a resident physician but do not require an immediate bedside evaluation by the entire medical team. While there are some objective criteria for activating priority assessments, unlike triggers, priority assessments can also be activated subjectively if a member of the team is concerned about the patient. The criteria for activating each type of expedited evaluation are outlined in Figure 1.

Our EHR sorts patients waiting for a physician to assign themselves by the following criteria:

1. Triggers, sorted with the oldest on top
2. Priority assessments, sorted by most recently to least recently moved to the top
3. ESI levels 1 and 2 who have not been claimed in > 1 hour, sorted by longest to shortest wait time
4. Patients in a treatment room, sorted by longest time in room to shortest
5. Patients in the ambulance waiting area, sorted by longest to shortest wait time
6. Waiting room patients, sorted by longest to shortest wait time

Resident physicians are instructed to assign themselves to patients in the order in which they appear on the list of patients to be seen to avoid skipping over potentially ill patients. In our ED, physician teams are not confined to specific geographical zones or room numbers and can see patients throughout the department in the appropriate order as determined by their acuity. This also means that patients can be evaluated while in the waiting room, where they can receive limited interventions such as lab draws and imaging that does not require intravenous contrast. Therefore, we evaluated “time-to-physician” rather than “time in waiting room” to more accurately measure patient wait times.

### Data Analysis

This was a retrospective study of all ED visits from July 2021–June 2023 in our tertiary-care academic medical center with a Level I Trauma and Comprehensive Stroke Center designations and an annual census over 50,000 visits. Our hospital does not have pediatric services; thus, patients < 18 years of age presenting to the ED are either transferred to a pediatric facility or evaluated at our facility on a case-by-case basis. All data were abstracted from the EHR by a single abstractor using best practices outlined by Worster and colleagues,^20^ which included pre-defined inclusion and exclusion criteria, variable definition prior to abstraction, and institutional review board approval (Protocol #: 2023P000990). We evaluated time-to-physician, defined as the time between a patient arriving to the ED and a physician claiming that patient in the EHR, and physician-to-decision time defined as the time elapsed between a physician assigning themselves to a patient and a disposition being selected for the patient. We included only encounters with a disposition other than “admission from the ED,” “discharge from the ED,” or “ED observation.” Encounters with missing timestamps for their time-to-physician and/or physician-to-decision were excluded. No specific patient populations were excluded.

We also evaluated the frequency of triggers and priority assessments in that set of visits. As the total number of encounters was high (> 100,000) and the number excluded was low (1%), we did not employ any statistical methods to address missing data. We had two exposures, race and language, and four outcomes: 1) time-to-physician; 2) physician-to-decision time, 3) proportion of triggers; and 4) proportion of priority assessments. Each outcome was assessed considering the exposure of interest. A secondary analysis stratified the above analyses on disposition (admission, observation, or discharge).

Patient demographics including age, sex, race, ethnicity (Hispanic/Latino/a/x vs non-Hispanic/Latino/a/x), and primary language are reported by patients upon initial registration into our system and were obtained from the EHR. To avoid potential collinearity between race and ethnicity, these variables were analyzed in combination, and the following racial categories were used: “Hispanic/Latino/a/x”; “Black, non-Hispanic”; “White, non-Hispanic”; “Asian, non-Hispanic”; “American Indian/Alaska Native, non-Hispanic”; “Native Hawaiian/other Pacific Islander, non-Hispanic”; and “Other or Unknown.” Some research suggests that Hispanic/Latino/a/x respondents better identify with questions on race and Hispanic ethnicity when a one-question format is used given that many Hispanic/Latino/a/x patients do not relate to standard racial categories provided by the US Census.^21^–^23^ Language categories were classified as “English” and “other.” We excluded patients without available language data from the analysis regarding language.

With regard to demographics among groups, we compared continuous variables using a one-way ANOVA with a Bonferroni correction or a Student *t*-test, based on the number of groups. Categorical variables were compared between groups using a chi-squared test. For unadjusted hypothesis testing, we compared medians between racial/ethnic groups using a Kruskal-Wallis test and between language using a Wilcoxon rank-sum test. Differences in groups in terms of time-to-physician or physician-to-decision were estimated from quantile regressions controlling for age, ESI, and sex and were provided as adjusted median differences (aMD) with a 95% CI. Proportions of triggers were presented with 95% CI calculated using the binomial exact method, and we used logistic regressions controlling for age, ESI, and sex to compare differences in trigger rates with regression estimates provided as adjusted odds ratios (aOR) with a 95% CI. All analyses were then stratified by ED disposition to evaluate how potential differences in patient acuity affected results and the methods above were repeated on each stratum. A *P*-value of < .05 was a priori established to indicate statistical significance. All analyses were performed using Stata 18.5 (StataCorp, LLC, College Station, TX).^24^ We adhered to STROBE reporting guidelines for this study.^25^

## RESULTS

There was a total of 103,370 encounters during the selected time period, of which the following were excluded: 1,503 because of a missing door-to-physician time; two due to a negative door-to-physician time; and 9,640 due to a lack of decision time, leaving a total of 93,728 eligible patient encounters. (Encounters could have more than one exclusion.) We also excluded 56 encounters from the language analysis only for missing language. The mean age was 55.0 ± 20.9 years, 54.5% were female, and the median ESI was 3 (IQR 2, 3). Demographic information can be found in our supplemental tables. Patients of Black race had a median time-to-physician of 31 minutes compared to 24 minutes for White patients (aMD 3.2, 95% CI 2.4–3.9). Patients of Hispanic/Latino ethnicity had a median time-to-physician of 31 (aMD compared to White patients: 3.4, 95% CI 2.4–3.9). These differences in wait time remained even when we stratified the analysis by ED disposition. There was no statistically significant difference in median physician wait times between patients of White race and those of Asian, American Indian/Alaska Native, Native Hawaiian/Other Pacific Islander, or other/unknown/unspecified race. In terms of language, patients with a preferred language other than English had a median time-to-physician of 33 minutes compared to 25 in English-preferring patients (aMD 4.6, 95% CI:3.7–5.6) (Table 1).Patients of Black race had median physician-to-decision times of 228 minutes compared to 213 for White patients (aMD 15.0, 95% CI 12.0–17.9). Hispanic/Latino patients had a median physician-to-decision time of 233 minutes (aMD compared to White patients: 21.3, 95% CI 17.5–25.2). Patients of Asian race had shorter median physician-to-decision times when discharged (median difference: −9.2, 95% CI −14.8 to −3.6) as compared to White patients. Patients with other/unknown/unspecified race had significantly shorter physician-to-decision times that those of White race when admitted (median difference: −34.9, 95% CI −42.0 to −27.8 minutes) and longer when under observation (median difference: 24.0, 95% CI 4.9–43.1). Patients with a preferred language other than English had a median physician-to-disposition time 17.1 minutes longer (95% CI 13.6–20.7) compared to patients who preferred English (Table 2).We then evaluated the proportion of triggers and priority assessments in that same population (Tables 3 and 4). Black patients (aOR 0.88, 95% CI 0.82–0.95) were less likely to have a trigger activated, whereas patients with other/unknown/unspecified race (aOR 1.63, 95% CI 1.48–1.79) were more likely to have a trigger activated relative to White patients. There was no significant difference regarding language overall; however, non-English preferring patients who were eventually placed in observation (aOR 1.39, 95% CI 1.04–1.85) were significantly more likely to have a trigger called than English-preferring patients. Similarly, Black patients (aOR 0.72, 95% CI 0.67–0.77]), Hispanic/Latino patients (aOR 0.78, 95% CI 0.71–0.86), and non-English-preferring patients (aOR 0.85, 95% CI 0.78–0.92) were less likely to have a priority assessment called than White or English-speaking patients.

## DISCUSSION

Our study found that in an EHR system where ESI is not the only factor used in signaling patient acuity to physicians, Black, Hispanic, and non-English-preferring patients experienced delays in seeing a physician and in waiting for a disposition, regardless of the final disposition. While the adjusted median differences, which ranged from a few minutes to over half an hour for some populations, may seem small and of doubtful clinical significance, their aggregate effect over the ≈ 100,000 encounters studied translates into many hours of delays in initial evaluation and reassessments that could impact patient outcomes. The reasons behind these disparities are likely multifactorial, involving both interpersonal and structural factors. From a triage standpoint, implicit bias among healthcare physicians may influence triage decisions and the perceived urgency of a patient’s condition.^26^–^29^ Prior literature suggests that heightened time pressure and higher patient loads can exacerbate reliance on implicit biases,^30^–^33^ meaning that systemic factors such as ED crowding could be affecting physician decisions and heightening racial and ethnic disparities in wait times.

Structural factors such as limited access to outpatient care might also influence racial and ethnic disparities seen in our analysis, as patients without primary care or outpatient specialists may not have as much documentation about their medical conditions readily available in the EHR for a triage nurse or physician to review. Studies have shown that Black and Hispanic patients have lower use of outpatient services.^34^–^36^ Some patients may also lack the advocacy that outpatient medical teams can offer by requesting an admission on their behalf or by documenting specific concerns that the patients themselves may have difficulty conveying. This might lead to some patients undergoing more extensive workups given higher levels of uncertainty about their diagnosis and plan, which can delay their disposition.

Our analysis showed an association between LEP and longer wait times. Limited availability of interpreters can further exacerbate delays in patients with LEP. In fact, Wallbrecht and colleagues found that although there was no statistically significant difference in ED LOS between patients with LEP and English-speaking patients, when they stratified by interpreter vs no interpreter use, patients who required an interpreter did have longer LOS than patients with LEP who did not require an interpreter.^14^ In our department, we have the ability to request phone interpreters from our hospital interpreter services for certain languages commonly spoken in our ED. During off-business hours or for less common languages, we also have video and audio interpreters available via smart tablets. Given that the process of requesting an interpreter can take time—particularly during off hours or for less common languages—it is possible that our door-to-physician metric is falsely underestimating the time that non-English-preferring patients who required an interpreter waited to see a doctor. Similarly, interpreter wait times could be adversely affecting the physician-to-decision times, as physicians may delay communicating with these patients if an interpreter is not readily available.

Our analysis of urgent evaluations showed an association between Black race and a lower likelihood of trigger activation. It is possible that Black patients were less ill upon presentation to the ED. While criteria for triggers are meant to be as objective as possible, the role of racial bias should also be considered. For example, while stroke symptoms are part of the criteria for a trigger activation, prior literature shows that Black patients experience longer ED wait times and delayed treatment for stroke relative to White patients.^9^ Interestingly, non-English-language preferring patients who were placed in observation were significantly more likely to have a trigger called than English-preferring patients. The nature of this analysis did not allow us to evaluate the timing of triggers with respect to disposition, making it difficult to ascertain whether these were patients who had a trigger activation prior to their disposition being set or had triggers activated while they were in ED observation.

Our analysis also showed an association between Black race, Hispanic/Latino ethnicity, and LEP, and priority assessment activation. Although there are some objective criteria for priority assessments, they can also be activated subjectively; they are meant to give nurses and physicians the ability to have urgently evaluated a patient with a concerning history or presentation who doesn’t meet criteria for a trigger activation. Thus, priority assessments are more susceptible to bias as well as to the over-reliance on external historians and prior documentation available in the EHR previously discussed.

This study highlights the pervasive racial, ethnic, and language-based differences experienced by Black, Hispanic/Latino, and non-English-language preferring patients and underscores the need to develop solutions to address these disparities. Potential interventions to mitigate these disparities include implicit bias training and subsequent education on debiasing strategies.^37^,^38^ Another area of opportunity that remains to be explored is leveraging artificial intelligence models to aid in triage decisions. A scoping review of the use of artificial intelligence in ED by Tyler and colleagues found that machine-learning models used in triage can reduce under- and over-triage, alleviate the workload of medical staff, optimize resource allocation, predict patient disposition, and improve identification of critically ill patients.^39^ However, prior studies have shown racial and ethnic bias in currently available models.^40^

## LIMITATIONS

Our analysis did not incorporate other important variables in determining patient acuity and complexity during the triage process, such as patient comorbidities, initial vital signs, and chief complaint. While we did not account for differences in patient acuity beyond stratifying our analysis by ED disposition, other studies that have controlled for patient acuity have found that Black and Hispanic patients as well as patients with LEP are more likely to be jumped over in line by patients with similar acuity.^3^,^16^ In addition, even if Black, Hispanic, and LEP patients were presenting with lower acuity complaints, this would not necessarily explain why the physician-to-decision times are longer for these groups. It’s difficult to ascertain why 9,642 encounters had missing or nonsensical timestamps. An initial theory was that timestamps were missing because some of these patients were critically ill and required timely interventions that prevented timestamps from being adequately recorded. However, as outlined in Supplemental Table 4, 7% of excluded patients (vs 4% of excluded patients) had an ESI of 1, making it unlikely that being more critically ill was the leading factor behind the missing timestamps.

In our trigger analysis, we only evaluated patients who had a trigger activated; there might have been patients for whom triggers should have been activated who did not have one activated at all. In addition, the reason for trigger activation was beyond the scope of this analysis. We also did not evaluate for other variables that may be correlated with triage decisions, such as history of being undomiciled, insurance status and type, primary care physician or specialist within our system, or prior visits within our ED. For our patients with LEP, we were unable to determine whether they required an interpreter, as this data was not available at the encounter level. We also did not have ED census data available to us to control for crowding on a given date at a given time.

It is important to note that race, ethnicity, and preferred language data collected from EHRs can often be inaccurate and incomplete.^41^,^42^ Lastly, any adverse effects caused by delays in time-to-physician and physician-to-decision, as well as by any potential missed triggers, remain to be determined and could serve as the basis for future studies within our health system.

## CONCLUSION

This study evaluated racial, ethnic, and language-based disparities in delays seeing a physician, waiting for a disposition, and having an urgent evaluation in an ED where urgent evaluations rather than Emergency Severity Index scores alone are used to signal patient acuity to physicians. Black, Hispanic, and non-English-preferring patients experience delays in seeing a physician and in waiting for a disposition, regardless of their final disposition. There is an association with Black race and lower likelihood of trigger activation. There is also an association with Black race, Hispanic ethnicity, and LEP; and lower likelihood of having priority assessments activated. While the effects of these delays and potentially missed urgent evaluations remains to be determined, these findings underscore the need to develop additional mechanisms for mitigating biases in the triage process.

## Supplementary Information



## Figures and Tables

**Table 1:** Quantile regression of time to-physician, controlling for Emergency Severity Index, age, and sex.

	Adjusted median difference (95% CI) minutes	*P*-value	Unadjusted median (IQR) minutes
All dispositions			
Race			
White (n = 49,656)	reference		24 (9, 62)
Black (n = 21,356)	3.2 (2.4–3.9)	< .001	31 (11, 73)
Hispanic/Latino (n = 10,768)	3.4 (2.4–4.4)	< .001	31 (11, 72)
Asian (n = 5,763)	0.5 (−0.8–1.9)	.43	26 (9, 68)
American Indian/Alaska Native (n = 287)	2.6 (−3.1–8.3)	.37	28 (10, 67)
Native Hawaiian/other Pacific Islander (n = 80)	−0.3 (−11.0–10.4)	.96	27 (10, 56)
Other/Unknown/Not Specified (n = 5,818)	−0.7 (−2.0, 0.6)	.31	20 (8, 55)
Language			
English (n= 82,060)	reference		25 (9, 64)
Other (n = 11,612)	4.6 (3.7–5.6)	< .001	33 (12, 77)
Admitted from the ED			
Race (n = 35,352)			
White (n = 22,487)	reference		
Black (n = 6,019)	3.0 (1.8–4.2)	< .001	
Hispanic/Latino (n = 2,602)	2.0 (0.3–3.7)	.02	
Asian (n = 1,639)	2.0 (−0.1–4.1)	.06	
American Indian/Alaska Native (n = 111)	−1.0 (−8.9–6.9)	.80	
Native Hawaiian/other Pacific Islander (n = 27)	−2.0 (−17.9–13.9)	.81	
Other/unknown/not specified (n = 2,467)	−2.0 (−3.8, −0.2)	.03	
Language (n = 35,343)			
English (n = 31,070)	reference		
Other (n = 4,273)	3.0 (1.6–4.4)	< .001	
Discharged from the ED			
Race (n = 48,017)			
White (n = 21,468)	reference		
Black (n = 12,712)	2.4 (1.2–3.5)	< .001	
Hispanic/Latino (n = 7,167)	3.7 (2.3–5.1)	< .001	
Asian (n = 3,596)	0.3 (−1.6–2.2)	.76	
American Indian/Alaska Native (n = 137)	3.4 (−5.5–12.3)	.46	
Native Hawaiian/other Pacific Islander (n = 47)	−4.5 (−19.7–10.6)	.56	
Other/unknown/not specified (n = 2,890)	1.9 (−0.2–4.0)	.07	
Language (n = 47,974)			
English (n = 41,704)	reference		
Other (n = 6,270)	6.0 (4.6–7.4)	< .001	
Observation			
Race			
White (n = 701)	reference		
Black (n = 2625)	4.6 (2.3–7.0)	< .001	
Hispanic/Latino (n = 999)	3.7 (0.3–7.1)	.03	
Asian (n = 528)	0.4 (−4.1–4.8)	.88	
American Indian/Alaska Native (n = 39)	17.0 (1.3–32.7)	.03	
Native Hawaiian/other Pacific Islander (n = 6)	34.0 (−5.9–73.9)	.10	
Other/unknown/not specified (n = 461)	−2.7 (−7.5–2.1)	.27	
Language (n = 10,355)			
English (n = 9,286)	reference		
Other (n = 1,069)	4.0 (0.8–7.3)	.02	

*IQR*, interquartile range; *ED*, emergency department.

**Table 2:** Quantile regression of physician-to-decision time controlling for Emergency Severity Index, age, and sex.

	Adjusted median difference (95% CI) minutes	P-value	Unadjusted median (IQR) minutes
All dispositions			
Race (n = 93,728)			
White (n = 49,656)	Reference		213 (129, 328)
Black (n = 21,356)	15.0 (12.0–17.9)	< .001	228 (141, 343)
Hispanic/Latino (n = 10,768)	21.3 (17.5–25.2)	< .001	233 (148, 344)
Asian (n = 5,763)	−1.0 (−5.9–4.0)	.71	208 (127, 315)
American Indian/Alaska Native (n = 287)	19.3 (−1.6–40.1)	.07	232 (154, 334)
Native Hawaiian/other Pacific Islander (n = 80)	23.2 (−16.3–62.7)	.25	229 (143, 313)
Other/Unknown/Not specified (n = 5,818)	−21.0 (−26.0, −16.1)	< .001	183 (102, 301)
Language (n = 93,672)			
English (n = 82,060)	Reference		214 (130, 329)
Other (n = 11,612)	17.1 (13.6–20.7)	< .001	234 (146, 345)
Admitted from the ED			
Race (n = 35,352)			
White (n = 22,487)	Reference		
Black (n = 6,019)	20.8 (16.0–25.6)	< .001	
Hispanic/Latino (n = 2,602)	27.9 (21.4–34.8)	< .001	
Asian (n = 1,639)	6.6 (−1.8–15.1)	.12	
American Indian/Alaska Native (n = 111)	14.3 (−17.1–45.6)	.37	
Native Hawaiian/other Pacific Islander (n = 27)	36.6 (−26.9–100.0)	.26	
Other/unknown/not specified (n = 2,467)	−34.9 (−42.0, −27.8)	< .001	
Language (n = 35,343)			
English (n = 31,070)	Reference		
Other (n = 4,273)	18.5 (12.9–24.1)	< .001	
Discharged from ED			
Race (n = 48,017)			
White (n = 21,468)	reference		
Black (n = 12,712)	0.9 (−2.5–4.4)	.59	
Hispanic/Latino (n = 7,167)	9.8 (5.5–14.0)	< .001	
Asian (n = 3,596)	−9.2 (−14.8, −3.6)	< .01	
American Indian/Alaska Native (n = 137)	14.3 (−12.1–40.7)	.29	
Native Hawaiian/other Pacific Islander (n = 47)	10.8 (−34.2–55.8)	.64	
Other/unknown/not specified (n = 2,890)	−2.4 (−8.6–3.7)	.44	
Language (n = 47,974)			
English (n = 41,704)	reference		
Other (n = 6,270)	8.7 (4.5–13.0)	< .001	
Placed in ED observation			
Race (n = 10,359)			
White (n = 5,701)	reference		
Black (n = 2,625)	10.3 (1.0–19.6)	.03	
Hispanic/Latino (n = 999)	11.2 (−2.4–24.7)	.11	
Asian (n = 528)	−1.0 (−19.1–17.0)	.91	
American Indian/Alaska Native (n = 39)	7.7 (−55.4–70.8)	.81	
Native Hawaiian/other Pacific Islander (n = 6)	−15.2 (−175.6–145.3)	.85	
Other/unknown/not specified n = 461)	24.0 (4.9–43.1)	.01	
Language (n = 10,355)			
English (n = 9,286)	reference		
Other (n = 1,069)	21.6 (8.5–34.7)	< .01	

*IQR*, interquartile range; *ED*, emergency department.

**Table 3:** Logistic regression of proportion of triggers controlling for Emergency Severity Index, age, and sex.

	Proportion (95% CI)	aOR (95%)	P-value
All dispositions			
Race			
White (n = 49,656)	0.112 (0.109–0.114)	Reference	
Black (n = 21,356)	0.073 (0.070–0.077)	0.88 (0.82–0.95)	< .01
Hispanic/Latino (n = 10,768)	0.072 (0.067–0.077)	0.94 (0.85–1.04)	.22
Asian (n = 5,763)	0.087 (0.080–0.095)	1.07 (0.95–1.19)	.28
American Indian/Alaska Native (n = 287)	0.108 (0.075–0.150)	1.15 (0.74–1.79)	.53
Native Hawaiian/other Pacific Islander (n = 80)	0.100 (0.044–0.188)	1.15 (0.43–3.06)	.78
Other/unknown/not specified (n = 5,818)	0.191 (0.181–0.202)	1.63 (1.48–1.79)	< .001
Language			
English (n = 82,060)	0.103 (0.101–0.105)	Reference	
Other (n = 11,612)	0.094 (0.089–0.100)	1.00 (0.92–1.08)	.99
Admitted from the ED			
Race (n = 35,352)			
White (n = 22,487)	0.179 (0.174–0.184)	Reference	
Black (n = 6,019)	0.161 (0.143–0.171)	0.99 (0.90–1.08)	.79
Hispanic/Latino (n = 2,602)	0.157 (0.143–0.171)	0.93 (0.81–1.07)	.31
Asian (n = 1,639)	0.173 (0.155–0.192)	1.02 (0.87–1.19)	.80
American Indian/Alaska Native (n = 11)	0.162 (0.099–0.244)	1.14 (0.62–2.09)	.67
Native Hawaiian/other Pacific Islander (n = 27)	0.222 (0.086–0.423)	1.41 (0.43–4.66)	.57
Other/unknown/not specified (n = 2,467)	0.378 (0.359–0.397)	1.82 (1.62–2.04)	< .001
Language (n = 35,343)			
English (n = 31,070)	0.189 (0.185–0.193)	reference	
Other (n = 4,273)	0.179 (0.168–0.191)	0.94 (0.85–1.04)	.27
Discharged from ED			
Race (n = 48,017)			
White (n = 21,468)	0.053 (0.050–0.056)	reference	
Black (n = 12,712)	0.037 (0.034–0.040)	0.90 (0.79–1.03)	.12
Hispanic/Latino (n = 7,167)	0.042 (0.037–0.047)	1.02 (0.87–1.19)	.83
Asian (n = 3,596)	0.050 (0.043–0.057)	1.11 (0.92–1.35)	.27
American Indian/Alaska Native (n = 137)	0.088 (0.046–0.148)	1.56 (0.77–3.16)	.22
Native Hawaiian/other Pacific Islander (n = 47)	0.043 (0.005–0.145)	0.86 (0.13–5.67)	.88
Other/unknown/not specified (n = 2,890)	0.048 (0.041–0.057)	1.10 (0.89–1.36)	.38
Language (n = 47,974)			
English (n = 41,704)	0.048 (0.046–0.050)	reference	
Other (n = 6,270)	0.040 (0.035–0.045)	1.13 (0.97–1.33)	.12
Placed in ED observation			
Race (n = 10,359)			
White (n = 5,701)	0.067 (0.060–0.074)	reference	
Black (n = 2,625)	0.048 (0.040–0.057)	0.88 (0.70–1.12)	.31
Hispanic/Latino (n = 999)	0.070 (0.055–0.088)	1.18 (0.86–1.63)	.30
Asian (n = 528)	0.078 (0.056–0.104)	1.49 (1.02–2.19)	.04
American Indian/Alaska Native (n = 39)	0.026 (0.001–0.135)	0.50 (0.07–3.67)	.49
Native Hawaiian/other Pacific Islander (n = 6)	0.000 (0.000–0.459)	Unable to be assessed due to no triggers in this group	
Other/unknown/not specified (n = 461)	0.089 (0.065–0.119)	1.19 (0.77–1.84)	.44
Language (n = 10,355)			
English (n = 9,286)	0.062 (0.058–0.068)	Reference	
Other (n = 1,069)	0.076 (0.061–0.093)	1.39 (1.04–1.85)	.02

*CI*, confidence interval; *aOR*, adjusted odds ratio; *ED*, emergency department.

**Table 4:** Logistic regression of proportion of priority assessments controlling for Emergency Security Index, age, and sex.

	Proportion (95% CI)	aOR (95% CI)	P-value
Race			
White (n = 49,656)	0.088 (0.086–0.090)	reference	
Black (n = 21,356)	0.050 (0.047–0.053)	0.72 (0.67–0.77)	< .001
Hispanic/Latino (n = 10.768)	0.050 (0.046–0.054)	0.78 (0.71–0.86)	< .001
Asian (n = 5,763)	0.067 (0.061–0.074)	0.93 (0.83–1.04)	.18
American Indian/Alaska Native (n = 287)	0.073 (0.046–0.110)	0.85 (0.53–1.34)	.48
Native Hawaiian/other Pacific Islander (n = 80)	0.050 (0.014–0.123)	0.81 (0.28–2.29)	.69
Other/unknown/not specified (n = 5,818)	0.079 (0.072–0.086)	1.05 (0.94–1.17)	.37
Language			
English (n = 82,060)	0.075 (0.073–0.077)	reference	
Other (n = 11,612)	0.060 (0.056–0.064)	0.85 (0.78–0.92)	< .001
Admitted from the ED			
Race (n = 35,352)			
White (n = 22,487)	0.117 (0.113–0.121)	reference	
Black (n = 6,019)	0.089 (0.081–0.096)	0.82 (0.74–0.90)	< .001
Hispanic/Latino (n = 2,602)	0.085 (0.075–0.097)	0.79 (0.68–0.92)	< 0.01
Asian (n = 1,639)	0.110 (0.095–0.126)	0.96 (0.81–1.13)	.60
American Indian/Alaska Native (n = 111)	0.108 (0.057–0.181)	1.08 (0.58–2.02)	.81
Native Hawaiian/other Pacific Islander (n = 27)	0.074 (0.009–0.243)	0.73 (0.16–3.25)	.68
Other/unknown/not specified (n = 2,467)	0.116 (0.104–0.129)	1.08 (0.94–1.24)	.28
Language (n = 35,343)			
English (n = 31,070)	0.110 (0.107–0.114)	reference	
Other (n = 4,273)	0.100 (0.091–0.110)	0.93 (0.83–1.04)	.21
Discharged from ED			
Race (n = 48,017)			
White (n = 21,468)	0.056 (0.053–0.059)	reference	
Black (n = 12,712)	0.031 (0.028–0.034)	0.68 (0.60–0.77)	< .001
Hispanic/Latino (n = 7,167)	0.034 (0.030–0.039)	0.78 (0.67–0.90)	< .01
Asian (n = 3,596)	0.048 (0.041–0.056)	1.01 (0.85–1.20)	.95
American Indian/Alaska Native (n = 137)	0.044 (0.016–0.093)	0.68 (0.29–1.59)	.38
Native Hawaiian/other Pacific Islander (n = 47)	0.021 (0.001–0.113)	0.52 (0.07–3.99)	.53
Other/unknown/not specified (n = 2,890)	0.042 (0.035–0.050)	0.90 (0.74–1.10)	.30
Language (n = 47,974)			
English (n = 41,704)	0.046 (0.044–0.048)	reference	
Other (n = 6,270)	0.033 (0.029–0.038)	0.81 (0.70–0.95)	< .01
Placed in ED observation			
Race (n = 10,359)			
White (n = 5,701)	0.098 (0.090–0.106)	reference	
Black (n = 2,625)	0.058 (0.049–0.067)	0.62 (0.51–0.75)	< .001
Hispanic/Latino (n = 999)	0.071 (0.056–0.089)	0.80 (0.61–1.04)	.10
Asian (n = 528)	0.066 (0.047–0.091)	0.66 (0.46–0.94)	.02
American Indian/Alaska Native (n = 39)	0.077 (0.016–0.209)	0.68 (0.21–2.27)	.54
Native Hawaiian/other Pacific Islander (n = 6)	0.167 (0.004–0.641)	2.53 (0.26–24.21)	.42
Other/unknown/not specified (n = 461)	0.108 (0.082–0.140)	1.30 (0.94–1.78)	.11
Language (n = 10,355)			
English (n = 9,286)	0.087 (0.081–0.093)	reference	
Other (n = 1,069)	0.056 (0.043–0.072)	0.62 (0.47–0.82)	< .01

*CI*, confidence interval; *aOR*, adjusted odds ratio; *ED*, emergency department.
