# Supplementary material for: Emergency Department Wait Times for Urgent Evaluation by Race, Ethnicity, and Language: A Single-center Retrospective Study
Source: West J Emerg Med. 2025 Sep 20;26(5):1232–43. doi: 10.5811/westjem.43480 (PMC12591622; doi:10.5811/westjem.43480)
Supplement: Supplementary file 1 [file wjem-26-1232-s001.docx]

**Supplemental Table 1.** Patient age, sex, and Emergency Severity Index by disposition.

|  | All (N = 93,728) | Admitted  (n = 35,352) | Observation  (n = 10.359) | Discharged  (n = 48,017) | *P*-value |
| --- | --- | --- | --- | --- | --- |
| Age, mean ±SD | 55.0±20.9 | 64.7±17.5 | 56.1±21.1 | 47.6±20.1 | < .001 |
| Female sex, n (%) | 51,121 (54) | 17,744 (50) | 5326 (51) | 28,051 (58) | < .001 |
| ESI, n (%) |  |  |  |  |  |
| 1 | 3,536 (4) | 2,685 (8) | 246 (2) | 605 (1) | < .001 |
| 2 | 36,943 (39) | 19.944 (56) | 5,357 (52) | 11,642 (24) |  |
| 3 | 47,932 (51) | 12,618 (36) | 4,559 (44) | 30,755 (64) |  |
| 4 | 5,212 (6) | 103 (0) | 195 (2) | 4,914 (10) |  |
| 5 | 105 (0) | 2 (0) | 2 (0) | 101 (0) |  |

*ESI,* Emergency Severity Index.

**Supplemental Table 2.** Patient age, sex, and Emergency Severity Index by race.

|  | American Indian/ Alaska Native  (n = 287) | Asian (n = 5,763) | Black  (n = 21,356) | Hispanic/Latino (n = 10.768) | Native Hawaiian/Pacific Islander  (n = 80) | Other/ Unknown (n = 5,818) | White  (n = 49,656) | *P*-value |
| --- | --- | --- | --- | --- | --- | --- | --- | --- |
| Age, mean ± SD | 53.4±17.6 | 47.4±22.9 | 53.5±19.3 | 48.4±18.5 | 45.8±20.9 | 50.8±21.7 | 58.5±20.9 | <.001 |
| Female sex, n (%) | 142 (49) | 3202 (56) | 13,078 (61) | 6349 (59) | 46 (58) | 2929 (50) | 25,375 (51) | <.001 |
| ESI, n (%) |  |  |  |  |  |  |  |  |
| 1 | 9 (3) | 154 (3) | 542 (3) | 289 (3) | 4 (5) | 605 (10) | 1,933 (4) | <.001 |
| 2 | 123 (43) | 2.067 (36) | 6,971 (33) | 3,208 (30) | 23 (29) | 2,205 (38) | 22,346 (45) |  |
| 3 | 147 (51) | 3,048 (53) | 12,177 (57) | 6,442 (60) | 45 (56) | 2,598 (45) | 23,475 (47) |  |
| 4 | 8 (3) | 482 (8) | 1,634 (8) | 817 (8) | 8 (10) | 399 (7) | 1864 (4) |  |
| 5 | 0 (0) | 12 (0) | 32 (0) | 12 (0) | 0 (0) | 11 (0) | 38 (0) |  |

*ESI,* Emergency Severity Index.

**Supplemental Table 3.** Patient age, sex, and Emergency Severity Index by preferred language.

|  | English (n = 82,060) | Non-English (n = 11,612) | *P*-value |
| --- | --- | --- | --- |
| Age, mean ± SD | 54.0±20.9 | 62.0±19.3 | <. 001 |
| Female sex, n (%) | 44,360 (54) | 6,734 (58) | < .001 |
| ESI, n (%) |  |  |  |
| 1 | 3,141 (4) | 393 (3) | < .001 |
| 2 | 32,842 (40) | 4,077 (35) |  |
| 3 | 41,465 (51) | 6,440 (55) |  |
| 4 | 4,514 (6) | 695 (6) |  |
| 5 | 98 (0) | 7 (0) |  |

*ESI,* Emergency Severity Index.

**Supplemental Table 4.** Demographics of patients excluded due to missing timestamps.

|  | **Included (n = 93,728)** | **Excluded (n = 9,642)** | ***P*-value** |
| --- | --- | --- | --- |
| **Age, mean ± SD** | 55.0±20.9 | 50.6±20.6 | < .001 |
| **Female sex, n (%)** | 51,121 (55) | 4,854 (50) | < .001 |
| **ESI, n (%)** |  |  | < .001 |
| **1** | 3,536 (4) | 630 (7) |  |
| **2** | 36,943 (39) | 3,630 (38) |  |
| **3** | 47,932 (51) | 4,760 (49) |  |
| **4** | 5,212 (6) | 560 (6) |  |
| **5** | 105 (0) | 62 (1) |  |
| **Race, n (%)** |  |  | < .001 |
| **White** | 49,656 (53) | 5,287 (55) |  |
| **Black** | 21,356 (23) | 2,087 (22) |  |
| **Hispanic/Latino** | 10,768 (11) | 914 (9) |  |
| **Asian** | 5,763 (6) | 392 (4) |  |
| **American Indian/Alaska Native** | 287 (0) | 32 (0) |  |
| **Native Hawaiian/Other Pacific Islander** | 80 (0) | 7 (0) |  |
| **Other/Unknown/Not specified** | 5,818 (6) | 923 (10) |  |
| **Language, n (%)** |  |  |  |
| **English** | 82,060 (88) | 8,237 (85) |  |
| **Other** | 11,612 (12) | 765 (8) |  |
| **Unknown** | 56 (0) | 640 (7) |  |

*ESI,* Emergency Severity Index.
